# Circulating Matrix Metalloproteinases and Tissue Inhibitors of Metalloproteinases in Cardiac Amyloidosis

**DOI:** 10.1161/JAHA.112.005868

**Published:** 2013-04-24

**Authors:** Komei Tanaka, Eric E. Essick, Gheorghe Doros, Kahraman Tanriverdi, Lawreen H. Connors, David C. Seldin, Flora Sam

**Affiliations:** 1 Whitaker Cardiovascular Institute, Boston University School of Medicine, Boston, MA (K.T., E.E.E., K.T., F.S.); 2 Cardiovascular Section and Evans Department of Medicine, Boston University School of Medicine, Boston, MA (F.S.); 3 Alan and Sandra Gerry Amyloid Research Laboratory in the Amyloid Treatment and Research Program, Boston University School of Medicine, Boston, MA (L.H.C., D.C.S.); 4 Department of Biostatistics, Boston University School of Public Health, Boston, MA (G.D.)

**Keywords:** cardiac amyloidosis, immunoglobulin light chain, matrix metalloproteinases, tissue inhibitors of metalloproteinases, transthyretin

## Abstract

**Background:**

Cardiac amyloidosis due to amyloid fibril deposition in the heart results in cardiomyopathy (CMP) with heart failure (HF) and/or conduction disturbances. Immunoglobulin light chain–related CMP (AL‐CMP) features rapidly progressive HF with an extremely poor prognosis compared with a CMP due to the deposition of mutant (ATTR) amyloidosis or wild‐type (senile systemic amyloidosis, SSA) transthyretin (TTR) proteins. Amyloid fibril deposition disrupts the myocardial extracellular matrix (ECM) homeostasis, which is partly regulated by matrix metalloproteinases (MMPs) and their tissue inhibitors (TIMPs). We therefore tested the hypothesis that circulating levels of MMPs and TIMPs in patients with AL‐CMP and TTR‐related CMP (TTR‐CMP) are dissimilar and indicative of cardiac amyloid disease type.

**Methods and Results:**

Fifty AL‐CMP patients were compared with 50 TTR‐CMP patients (composed of 38 SSA and 12 ATTR patients). Clinical and laboratory evaluations including echocardiography were performed at the initial visit to our center and analyzed. Serum MMP‐2, MMP‐9, TIMP‐1, and TIMP‐2 levels were determined by ELISA. Compared with TTR‐CMP patients, AL‐CMP patients had higher levels of brain natriuretic peptide (BNP), troponin I (TnI), MMP‐2, TIMP‐1, and MMP‐2/TIMP‐2 ratio, despite less left ventricular (LV) hypertrophy and better preserved LV ejection fraction. Mortality was worse in AL‐CMP patients than in TTR‐CMP patients (log‐rank *P*<0.01). MMP‐2/TIMP‐2 plus BNP and TnI showed the highest discriminative ability for distinguishing AL‐CMP from TTR‐CMP. Female sex (HR, 2.343; *P*=0.049) and BNP (HR, 1.041; *P*<0.01) were predictors for mortality for all patients with cardiac amyloidoses. Only BNP was a predictor of death in AL‐CMP patients (HR, 1.090; *P*<0.01). There were no prognostic factors for all‐cause death in TTR‐CMP patients.

**Conclusions:**

Circulating concentrations of MMPs and TIMPs may be useful in differentiating patients with AL‐CMP from those with TTR‐CMP, resulting in earlier diagnostic vigilance, and may add prognostic information. In addition to an elevated BNP level, female sex increased the risk of death in patients with cardiac amyloidoses.

## Introduction

Cardiac amyloidosis is a rare disease characterized by amyloid fibril deposition in the heart, resulting in a cardiomyopathy (CMP) that may manifest clinically with heart failure (HF) or conduction disturbances.^1–3^ Immunoglobulin light chain (LC) is the deposited amyloid protein in primary (AL) amyloidosis. Wild‐type or mutant forms of the plasma protein transthyretin (TTR) are amyloidogenic in senile systemic amyloidosis (SSA) or familial TTR (ATTR) amyloidosis. AL, SSA, and ATTR amyloidoses may manifest with cardiac involvement; but the clinical presentation is dependent on the type of precursor protein, and the outcome of each is quite different.^2,4^ AL‐related CMP (AL‐CMP) features rapidly progressive HF with an extremely poor prognosis and a median survival of <6 months when untreated.^3^ HF in SSA and ATTR‐related CMP is usually less severe and progresses more slowly than AL‐CMP. TTR‐CMP has a better prognosis than AL‐CMP.^5^ In some cases, the delay in obtaining a bone marrow biopsy for diagnosis invariably results in therapeutic delay that may prove fatal in AL‐CMP; thus, a noninvasive measurement to assist clinicians in selecting a bone marrow biopsy sooner may be useful.

Deposition of amyloid fibrils in the heart results in disruption of the myocardial extracellular matrix (ECM).^6–7^ The Myocardial ECM homeostasis and composition are determined, in part, by collagen degradation that is under the control of matrix metalloproteinases (MMPs), a family of proteolytic enzymes, and their tissue inhibitors (TIMPs).^8^ In nonamyloid heart disease, such as idiopathic dilated cardiomyopathy, ischemic heart disease, or hypertensive heart disease, elevated MMPs and TIMPs are associated with adverse cardiac remodeling, and dysfunction in clinical HF and CMP.^8–11^ We previously demonstrated increased MMP‐9 and TIMP‐1 expression in the myocardium of a small cohort of patients with AL‐CMP.^7^ Moreover, we also reported that in AL‐CMP, despite comparable left ventricular (LV) hypertrophy (LVH) with TTR‐CMP, there were higher circulating serum MMP‐9 and TIMP‐1 levels, which were associated with echocardiographic‐derived measures of impaired diastolic function.^7^ These findings suggest that LC deposition in the heart alters ECM homeostasis, activates the degradation system, and contributes to the pathogenesis of AL‐CMP. The disparate clinical manifestations of AL‐CMP and TTR‐CMP may be determined, in part, by variations in MMP and TIMP expression, which are dependent on amyloid protein being deposited in the heart. We therefore sought to determine whether circulating levels of MMPs and TIMPs in a new, larger cohort of cardiac amyloidosis patients would allow us to differentiate between patients with AL‐CMP and those with TTR‐CMP and perhaps aid in further clinical management.

## Methods

### Patient Data Collection

One hundred nonconsecutive patients were selected for study. These patients had biopsy‐proven amyloidosis featuring CMP and available clinical, laboratory, and echocardiographic data as well as serum samples obtained at initial visit to our center. All patients were >59 years of age and grouped according to amyloid disease type. Two study groups were analyzed; 50 patients with AL‐CMP were compared with 50 patients with TTR‐CMP in a combined group of 38 SSA and 12 ATTR patients. Clinical and laboratory evaluations were performed in the Amyloid Treatment and Research Program at Boston Medical Center between August 1996 and July 2010. The majority of the study patients (>80%) were recruited into the study within the last 5 years. The Boston University Medical Center Institutional Review Board approved the study, and written informed consent was obtained from all subjects. Clinical and laboratory evaluations, including a medical history, physical examination, routine blood test, routine urine test, chest radiography, electrocardiography (ECG), and echocardiography, were performed at the first visit to our clinic. All subjects had biopsy‐proven amyloidosis by positive Congo red staining of tissue specimens. Amyloid cardiac involvement was determined by a history of HF, low voltage on ECG, LVH on echocardiogram in the absence of a history of hypertension or valvular disease, or by an endomyocardial biopsy specimen that demonstrated amyloid deposits. Endomyocardial biopsy was obtained in 23 AL, 6 ATTR, and 32 SSA patients. Amyloid type was determined by immunohistochemical and biochemical analyses. Serum and urine immunofixation electrophoresis, free LC measurements, and bone marrow examination were performed in all subjects to identify a plasma cell dyscrasia. AL amyloidosis was diagnosed by the histopathological identification of monoclonal plasma cells in the bone marrow and by the detection of monoclonal LC in the serum and/or urine. Subjects in whom AL amyloidosis was excluded underwent testing for TTR‐related amyloidosis by immunohistochemical analysis of a biopsy, isoelectric focusing of serum to detect the presence of a mutant TTR, and direct DNA sequence analysis of all 4 coding exons of the *TTR* gene.^12–13^ ATTR amyloidosis was diagnosed with evidence of a pathological TTR mutation and SSA when only wild‐type alleles were present. All ATTR patients were African Americans with the V122I mutation.

### Echocardiography

Two‐dimensional (2D) echocardiography was performed using the GE VingMed Vivid FiVe Echocardiography System (GE Vingmed, Milwaukee, WI) with a 2.5‐MHz phased‐array transducer as previously described.^7^ Echocardiograms were performed and analyzed in a blinded manner. LV ejection fraction (LVEF) was calculated using the modified Simpson's rule, and measurements of systolic and diastolic chamber dimensions and wall thickness were obtained from 2D imaging according to the recommendations of the American Society of Echocardiography.^14^ The standard cube formula was used to calculate LV mass.^15^

### Biomarker Analysis

Blood samples were obtained at the initial visit to our center and stored at −80°C. Levels of brain natriuretic peptide (BNP), using the ADVIA Centaur assay (Siemens Healthcare Diagnostics), and troponin I (TnI) were measured as part of routine laboratory testing. Serum MMP‐2, MMP‐9, TIMP‐1, and TIMP‐2 levels were measured in duplicate with commercially available ELISA kits from GE Healthcare (Little Chalfont, Buckinghamshire, UK) and R&D Systems (Minneapolis, MN).

### Follow‐Up

Subjects were followed up annually in the Amyloid Treatment and Research Program at Boston Medical Center. For patients who could not visit the Amyloid Treatment and Research Program, their clinical course was monitored by telephone and/or by contacting referring physicians. The end point of follow‐up was a composite of all‐cause death.

### Statistical Analysis

Continuous variables are described as mean±standard deviation or median (interquartile range [IQR]). Categorical variables are described as number of patients. Comparisons between the AL‐CMP and TTR‐CMP groups were evaluated by the Student *t* test for continuous variables or the chi‐square test for categorical variables. Survival curves were estimated by Kaplan–Meier estimator. The Pearson product‐moment correlation analysis was used to evaluate the correlation between echocardiographic parameters, MMPs, TIMPs, and the ratio of MMPs to TIMPs. Predictive accuracy of BNP, TnI, MMP‐2, MMP‐9, TIMP‐1, TIMP‐2, MMP‐9/TIMP‐1, MMP‐2/TIMP‐2, and the combination of multiple biomarkers for differentiating between AL‐CMP and TTR‐CMP patients were evaluated by the area under the receiver operator characteristic (ROC) curve (AUC, or c‐statistics). Potential risk factors for the incidence of all‐cause death were evaluated by Cox proportional hazards regression analysis. The proportional hazards assumption was tested by a Kolmogorov‐type supremum test based on 1000 simulations and incorporated into the ASSESS statement of PROC PHREG.^16^ There was no significant evidence in these data that the proportional hazards assumption did not hold for the examined variables. Covariates were chosen on the basis of biological and statistical significance. Statistical significance relied on statistical tests for differences between the AL‐CMP and TTR‐CMP. Potential confounders were entered into a model, and then a backward elimination was conducted with *P*‐value criteria for retention set at 0.20. A value of *P*<0.05 was considered statistically significant. All analyses were conducted using SAS software version 9.2 (SAS Institute Inc.).

## Results

### Patient Characteristics

One hundred amyloidosis patients with cardiac involvement were included in the study. The patient study groups were AL‐CMP (n=50) and TTR‐CMP (n=50: 38 SSA and 12 ATTR). Clinical characteristics of each group at first visit to the Amyloid Treatment and Research Program are summarized in Table 1. Patients with AL‐CMP were significantly younger than those with TTR‐CMP. More AL‐CMP patients were female (38% versus 14%, *P*<0.01) and white (86% versus 58%, *P*<0.01) versus TTR‐CMP patients. Patients with AL‐CMP had worse functional status, as represented by higher New York Heart Association functional class, than those with TTR‐CMP (*P*<0.01). Systolic blood pressure was lower in AL‐CMP than in TTR‐CMP patients (*P*=0.025). BNP levels (670 [IQR, 388 to 1350] versus 395 [IQR, 252 to 521] pg/mL) and TnI levels (0.384±0.499 versus 0.186±0.100 ng/mL) were significantly higher in AL‐CMP compared with TTR‐CMP patients. Both AL‐CMP and TTR‐CMP patients had prolonged QRS on ECG, indicating underlying conduction disease. Diuretic use (loop or K^+^‐sparing diuretics) was similar in both groups of patients.

**Table 1.** Clinical Characteristics: AL‐CMP Patients Versus TTR‐CMP Patients

	AL‐CMP (n=50)	TTR‐CMP (n=50)	*P* Value
Race			
White, %	43 (86%)	29 (58%)	<0.01
Clinical			
Age, y	71.0±5.7	73.8±5.2	=0.010
Sex (female/male)	19/31	7/43	<0.01
Body mass index, kg/m^2^	26.5±4.2	27.0±4.0	=0.544
Body surface area, m^2^	1.83±0.21	1.94±0.20	<0.01
NYHA functional class	3.0±1.0	2.4±1.1	<0.01
Hemodynamics			
Systolic blood pressure, mm Hg	112.9±20.3	121.4±15.3	=0.025
Diastolic blood pressure, mm Hg	70.3±9.7	74.1±8.2	=0.045
Pulse rate, beats/min	82.6±15.5	73.9±12.5	<0.01
ECG			
QRS duration (ms) on ECG	104±29	115±33	=0.074
Biomarkers			
Hemoglobin, g/dL	12.9±1.7	13.3±1.7	=0.221
MDRD eGFR, mL/min per 1.73 m^2^	58.1±27.9	63.0±21.8	=0.334
C‐reactive protein, mg/dL	0.83±0.90	0.86±1.15	=0.883
BNP,* pg/mL	670.0 (388.0 to 1350.0)*	395.0 (252.0 to 521.0)*	=0.019*
TnI, ng/mL	0.384±0.499	0.186±0.1	=0.031

Data are expressed as mean±standard deviations for continuous variables or numbers for categorical variables. AL indicates immunoglobulin light chain amyloidosis; CMP, cardiomyopathy; TTR, transthyretin; NYHA, New York Heart Association; ECG, electrocardiogram; MDRD eGFR, glomerular filtration rate by Modification of Diet in Renal Disease equation; BNP, brain natriuretic peptide; TnI, troponin I.

* BNP displayed as median with IQR (interquartile range).

### LV Structure and Function

Echocardiographic data are summarized in Table 2. Although marked concentric LVH and increased LV mass were present in the both groups, these features were less pronounced in the AL‐CMP group compared with the TTR‐CMP group. For example, LV mass was 223.0±68.4 versus 291.7±75.1 g (*P*<0.001). Similarly, the ratio of LV mass/BSA was also greater in TTR‐CMP patients. Despite the increased LVH, LV diameter was larger in hearts with non‐LC amyloid deposition, as shown by the increased LV end‐systolic diameter in the TTR‐CMP group compared with the AL‐CMP group (3.12±0.69 versus 3.40±0.62 cm, *P*=0.047). LVEF in AL‐CMP patients was relatively preserved compared with the depressed LVEF in TTR‐CMP patients (46.2±15.5% versus 39.5±14.7%, *P*=0.037). Relative wall thickness was similar between AL‐CMP and TTR‐CMP patients.

**Table 2.** Echocardiographic Parameters: AL‐CMP Patients Versus TTR‐CMP Patients

	AL‐CMP (n=50)	TTR‐CMP (n=50)	*P* Value
Left atrium diameter, cm	4.17±0.70	4.29±0.52	=0.351
Interventricular septum wall thickness, cm	1.43±0.27	1.65±0.30	<0.001
LV posterior wall thickness, cm	1.43±0.23	1.65±0.29	<0.001
LV end‐diastolic diameter, cm	4.05±0.73	4.22±0.60	=0.244
LV end‐systolic diameter, cm	3.12±0.69	3.40±0.62	=0.047
LV ejection fraction, %	46.2±15.5	39.5±14.7	=0.037
LV mass, g	223.0±68.4	291.7±75.1	<0.001
LV mass/BSA, g/m^2^	120.2±34.3	153.0±38.8	<0.001
RWT	0.73±0.22	0.81±0.26	=0.140

Data are expressed as mean±standard deviations. AL indicates immunoglobulin light chain amyloidosis; CMP, cardiomyopathy; TTR, transthyretin; LV; left ventricular, BSA; body surface area; RWT; relative wall thickness.

### Outcome

**Figure 1.**
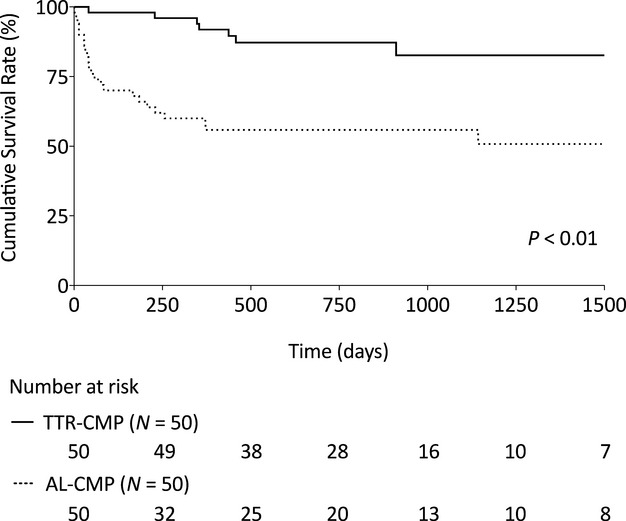
Kaplan–Meier survival curve. Cumulative survival rate in AL‐CMP patients and TTR‐CMP patients. AL indicates immunoglobulin light chain amyloidosis; CMP, cardiomyopathy; TTR, transthyretin.

Median duration of follow‐up was 453 days (IQR, 56 to 1025 days) in AL‐CMP patients and 788 days (IQR, 458 to 1090 days) in TTR‐CMP patients. The 1‐year survival rate was 60% in AL‐CMP patients and 92% in TTR‐CMP patients. Twenty‐three patients with AL‐CMP and 10 with TTR‐CMP died from any cause during the follow‐up period (Table 3). The cumulative survival rate in the AL‐CMP group was significantly lower than that in the TTR‐CMP group (log‐rank *P*<0.01; Figure 1).

**Table 3.** Causes of Death in Cardiac Amyloidosis

23 AL‐CMP patients (2 from malignant arrhythmia, 1 cardiovascular death, 1 hepatic failure, 8 severe heart failure, 1 sepsis, 7 sudden death, and 3 unknown)
10 TTR‐CMP patients (4 from severe heart failure, 1 malignancy, and 5 unknown)

AL indicates immunoglobulin light chain amyloidosis; CMP, cardiomyopathy; TTR, transthyretin.

### Circulating MMPs and TIMPs

**Figure 2.**
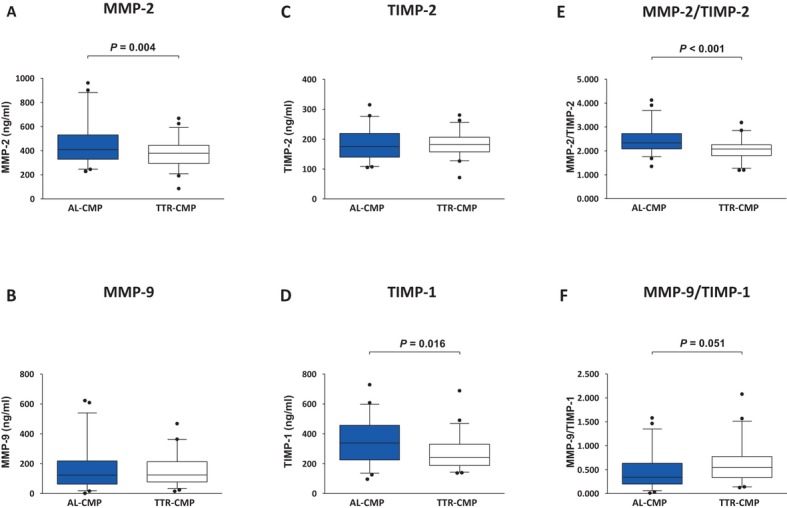
Circulating MMPs, TIMPs, and MMP/TIMP levels in cardiac amyloidosis patients. A, MMP‐2; B, MMP‐9; C, TIMP‐2; D, TIMP‐1; E, MMP‐2/TIMP‐2; F, MMP‐9/TIMP‐1. Box‐whisker plots show distributions (whiskers, 5% to 95%; box, 25% to 50% to 75%); n=50/group. MMP indicates matrix metalloproteinase; TIMP, tissue inhibitor of MMP; AL, immunoglobulin light chain amyloidosis; CMP, cardiomyopathy; TTR, transthyretin.

MMP‐2 levels in the AL‐CMP group (451.3±176.2 ng/mL) were significantly higher than in the TTR‐CMP group (376.4±112.4 ng/mL, *P*=0.004; Figure 2A). Interestingly, there was no significant difference in MMP‐9 levels between AL‐CMP (159.9±140.0 ng/mL) and TTR‐CMP (153.2±96.5 ng/mL; Figure 2B) patients. Similarly, TIMP‐2 levels were comparable between AL‐CMP (183.2±51.3 ng/mL) and TTR‐CMP (182.5±40.5 ng/mL; Figure 2C) patients. However, TIMP‐1 levels in the AL‐CMP group (347.1±146.4 ng/mL) were significantly higher compared with those in the TTR‐CMP group (269.2±111.3 ng/mL, *P*=0.016; Figure 2D). The ratio of MMP‐2 to TIMP‐2 (MMP‐2/TIMP‐2) was significantly higher in AL‐CMP than in TTR‐CMP (*P*<0.001; Figure 2E). The ratio of MMP‐9 to TIMP‐1 (MMP‐9/TIMP‐1) was not significantly different between the AL‐CMP and TTR‐CMP groups (*P*=0.051; Figure 2F). Differences in these circulating levels of MMPs and TIMPs were adjusted for age, sex, and race.

### Diagnostic Markers That Distinguish AL‐CMP and TTR‐CMP Groups

**Figure 3.**
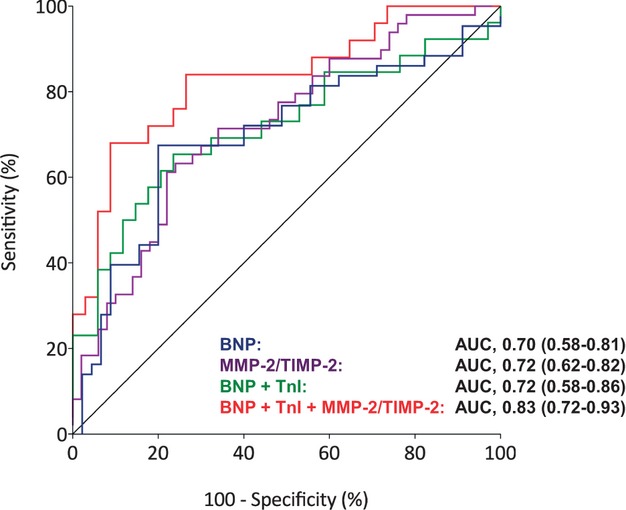
ROC curve. Predictive accuracy for differentiating between patients with AL‐CMP and TTR‐CMP. Areas under the ROC curve: BNP, 0.70 (95% CI, 0.58 to 0.81); MMP‐2/TIMP‐2, 0.72 (95% CI, 0.62 to 0.82); BNP+TnI, 0. 72 (95% CI, 0.58 to 0.86); BNP+TnI+MMP‐2/TIMP‐2, 0.83 (95% CI, 0.72 to 0.93). ROC indicates receiver operator characteristic; AL, immunoglobulin light chain amyloidosis; CMP, cardiomyopathy; TTR, transthyretin; BNP, brain natriuretic peptide; CI, confidence interval; MMP, matrix metalloproteinase; TIMP, tissue inhibitor of MMP; TnI, troponin I; AUC, area under the curve.

The power of single or multiple biomarkers to distinguish AL‐CMP from TTR‐CMP patients were evaluated using ROC analysis and c‐statistics. The AUC for TnI, MMP‐2, and TIMP‐1 was 0.59 (95% confidence interval [CI], 0.43 to 0.74), 0.60 (95% CI, 0.49 to 0.72), and 0.67 (95% CI, 0.56 to 0.78), respectively. The area under the ROC curve when using BNP alone, MMP‐2/TIMP‐2 alone, or the combination of BNP plus TnI was 0.70 (95% CI, 0.58 to 0.81), 0.72 (95% CI, 0.62 to 0.82), and 0.72 (95% CI, 0.58 to 0.86), respectively (Figure 3). However, the area under the ROC curve for MMP‐2/TIMP‐2 combined with BNP and TnI was 0.83 (95% CI, 0.72 to 0.93; Figure 3). The AUCs for the different biomarkers were also compared using the nonparameteric approach as described prevously.^17^ The inclusive model (BNP+TnI+MMP‐2/TIMP‐2) had the best predictive power versus BNP alone (*P*=0.09). Compared with BNP alone, the ratio of MMP‐2/TIMP‐2 (*P*=0.73) and the combination of BNP plus TnI (*P*=0.79) were not significant.

### Prognostic Factors for AL‐CMP and TTR‐CMP Groups

Prognostic factors for mortality in cardiac amyloidosis are shown in Table 4. Multivariate analysis using a Cox proportional hazards regression model with backward elimination showed that female sex (HR, 2.343; 95% CI, 1.001 to 5.481; *P*=0.049) and BNP (HR, 1.041; 95% CI, 1.011 to 1.071; *P*<0.01) were predictors of mortality in both groups of cardiac amyloidosis. Although not statistically significant, TIMP‐1 level (HR, 1.297; 95% CI, 0.982 to 1.713; *P*=0.067) showed a tendency to be a predictor. Prognostic factors for the incidence of all‐cause death in AL‐CMP alone are shown in Table 5. Multivariate analysis showed that only BNP (HR, 1.090; 95% CI, 1.041 to 1.141; *P*<0.01) was a predictor for all‐cause death in AL‐CMP. In this series of patients, there were no prognostic factors for the incidence of all‐cause death in TTR‐CMP. The smaller number of deaths in TTR‐CMP limited statistical power for the detection of prognostic indicators in TTR‐CMP.

**Table 4.** Prognostic Predictors for All‐Cause Death in Cardiac Amyloidosis (AL‐CMP and TTR‐CMP Patients)

	Univariate Hazard Ratio	*P* Value	Multivariate Hazard Ratio	*P* Value
Age*	0.977 (0.917 to 1.041)	0.473	—	—
Sex (female/male)	1.824 (0.873 to 3.811)	0.110	2.343 (1.001 to 5.481)	=0.049
NYHA functional class*	2.397 (1.486 to 3.866)	<0.01	—	—
BNP,§ pg/mL	1.043 (1.016 to 1.072)	<0.01	1.041 (1.011 to 1.071)	<0.01
TnI,§ ng/mL	1.084 (1.039 to 1.132)	<0.01	—	—
LV ejection fraction,* %	1.011 (0.986 to 1.037)	0.388	—	—
LV mass/BSA,* g/m^2^	0.994 (0.983 to 1.005)	0.294	—	—
MMP‐2,§ ng/mL	1.008 (0.988 to 1.030)	0.421	—	—
TIMP‐1,§ ng/mL	1.021 (0.998 to 1.043)	0.069	1.297 (0.980 to 1.055)	=0.067
MMP‐2/TIMP‐2§	1.019 (0.986 to 1.053)	0.270	—	—

AL indicates immunoglobulin light chain amyloidosis; CMP, cardiomyopathy; TTR, transthyretin; NYHA, New York Heart Association; BNP, brain natriuretic peptide; TnI, troponin I; LV; left ventricular; MMP, matrix metalloproteinase; TIMP, tissue inhibitor of MMP.

* Hazard ratio (95% confidence interval) per 1‐unit increment; ^†^hazard ratio per 100‐unit increment, ^‡^hazard ratio per 0.05‐unit increment,

§ hazard ratio per 10‐unit increment.

**Table 5.** Prognostic Predictors for All‐Cause Death in AL‐CMP Patients

	Univariate Hazard Ratio	*P* Value	Multivariate Hazard Ratio	*P* Value
Age*	1.004 (0.933 to 1.080)	0.923	—	—
Sex (female/male)	1.360 (0.596 to 3.107)	0.465	—	—
NYHA functional class*	1.822 (1.056 to 3.143)	0.031	—	—
BNP,§ pg/mL	1.062 (1.019 to 1.106)	<0.01	1.090 (1.041 to 1.141)	<0.01
TnI,§ ng/mL	1.045 (0.999 to 1.094)	0.055	—	—
LV ejection fraction,* %	1.000 (0.971 to 1.030)	0.988	—	—
LV mass/BSA,* g/m^2^	0.992 (0.977 to 1.007)	0.278	—	—
MMP‐2,§ ng/mL	1.001 (0.979 to 1.025)	0.903	—	—
TIMP‐1,§ ng/mL	1.011 (0.984 to 1.038)	0.433	—	—
MMP‐2/TIMP‐2§	0.992 (0.951 to 1.035)	0.713	—	—

AL indicates immunoglobulin light chain amyloidosis; CMP, cardiomyopathy; NYHA, New York Heart Association; BNP, brain natriuretic peptide; TnI, troponin I; LV; left ventricular; BSA, body surface area; MMP, matrix metalloproteinase; TIMP, tissue inhibitor of MMP.

* Hazard ratio (95% confidence interval) per 1‐unit increment; ^†^hazard ratio per 100‐unit increment, ^‡^hazard ratio per 0.05‐unit increment,

§ hazard ratio per 10‐unit increment.

## Discussion

In this unique data set, in a rare disease, the findings of the present study can be summarized as follows: (1) patients with AL‐CMP had higher levels of BNP, TnI, MMP‐2, TIMP‐1, and MMP‐2/TIMP‐2 ratio despite less LVH and relatively preserved LVEF compared with patients with TTR‐CMP; (2) AL‐CMP had a worse prognosis compared with TTR‐CMP; (3) MMP‐2/TIMP‐2 combined with BNP and TnI levels was highly discriminative for differentiating patients with AL‐CMP from those with TTR‐CMP at the initial visit to our center and better than BNP alone; (4) BNP and female sex were predictors of mortality in all cardiac amyloidosis, whereas only elevated BNP portended a worse prognosis in AL‐CMP.

### MMPs and TIMPs in Cardiac Amyloidosis

In both LV systolic or diastolic HF, regardless of etiology, changes in the ECM with myocardial collagen accumulation, collagen fibril disruption, myocyte loss, and altered spatial orientation of cells are seen.^8^ Myocardial ECM homeostasis is partly regulated by a balance between a family of proteolytic enzymes (MMPs) that degrade the ECM and their tissue inhibitors, the TIMPs.^8^ Distinct patterns of MMP and TIMP expression occur in the failing myocardium, and these patterns vary with the type and stage of disease.^8–11^ In a smaller study we showed that in a group of AL‐CMP patients higher levels of BNP, MMPs, and TIMPs correlated with echocardiographic measures of diastolic dysfunction, suggesting a relationship between LC proteins and ECM proteolytic activation.^7^ In the present, larger study, levels of MMP‐2 and TIMP‐1, and the MMP‐2/TIMP‐2 ratio (an index of net MMP‐2 activation) were increased in the AL‐CMP group. With LC misfolding and amyloid deposition in AL‐CMP, there is increased ECM degradation and disruption, as reflected by MMP‐2 and MMP‐2/TIMP‐2 upregulation. Similarly, our data suggest that with the upregulation of TIMP‐1 levels (which is the inhibitor of MMP‐9), net MMP‐9 activation may not play a role in AL‐CMP. These results suggest that ECM homeostasis is more altered in AL‐CMP than in TTR‐CMP patients.

Several types of MMPs are present in the heart, and the gelatinases (MMP‐2 and MMP‐9) have substrate affinity for denatured fibrillar collagen and basement membrane proteins. MMP‐2 and MMP‐9 are reported to be the key metalloproteinases in the heart that are increased in response to oxidative injury.^18–19^ Recent work showed that LC deposition alters the cellular redox state in cardiomyocytes, exerting negative inotropic effects and impairing excitation contraction coupling via increased oxidant stress.^20–21^ Thus, MMP‐2 and MMP‐9 and their inhibitors were the circulating levels that were selected for measurement in this study.

### Diagnostic Utility of MMPs and TIMPs in AL‐CMP and TTR‐CMP

Clinical criteria alone are inadequate to distinguish amyloid types in cardiac amyloidoses,^22^ and definitive typing relies on immunohistochemical and biochemical techniques (including mass spectrometry) performed on tissue biopsies.^23–24^ Other measures such a pressure–volume indices may assist in the diagnosis.^25^ Thus, noninvasive methods to distinguish AL‐CMP from TTR‐CMP would be informative. In this regard, 99mTc‐3,3‐diphosphono‐1,2‐propanodicarboxylic acid scintigraphy^26^ and cardiac MRI^27–28^ are useful in the workup of cardiac amyloidosis patients. Moreover, cardiac troponin and N‐terminal pro‐B‐type natriuretic peptide (NT‐pro‐BNP) have been proposed for use as prognostic markers in patients with AL amyloidosis.^29–31^ However, no biochemical marker is presently available for discriminating between AL‐CMP and TTR‐CMP.

In our study, BNP alone and the combination of BNP and TnI showed moderate utility in distinguishing patients with AL‐CMP from those with TTR‐CMP. Interestingly, MMP‐2/TIMP‐2 was equivalent to BNP alone as a differential diagnostic marker for AL‐CMP versus TTR‐CMP. Furthermore, MMP‐2/TIMP‐2 combined with BNP and TnI had the highest discriminating ability and the most predictive power (when compared with BNP alone). Thus, the marker MMP‐2/TIMP‐2 (especially when used in an inclusive model with BNP and TnI) may reduce the rate of misdiagnosis of AL‐CMP and TTR‐CMP and potentially allow for earlier diagnosis/treatment of AL‐CMP.

### Prognostic Utility of MMPs and TIMPs in Cardiac Amyloidosis

Factors (such as peak oxygen consumption, 6‐minute walk distance, and natriuretic peptides) are predictors of mortality in nonamyloid HF.^32–33^ The biomarkers NT‐proBNP, troponin T, and TnI have been used as measures of prognosis and response to therapy for AL amyloidosis.^31,34^ In our study BNP and female sex and possibly TIMP‐1 were predictors for all‐cause death in cardiac amyloidosis patients. Similar to the findings of Rapezzi et al,^35^ who found that male sex had a lower risk of death, we found that female sex portends a greater risk of dying in cardiac amyloidosis. Rapezzi et al also found that for long‐term outcome, the type of cardiac amyloidosis, that is, TTR‐CMP (specifically ATTR), was a favorable predictor of survival, and SSA predicted freedom from major cardiac events.^35^ Female sex as a risk was only seen in the combined cardiac amyloidoses and not the AL‐CMP group, likely because the numbers of patients were too small to detect a difference (although the majority of female patients were in the AL‐CMP group). Finally, follow‐up time differed between AL‐CMP and TTR‐CMP patients because AL‐CMP patients with have higher mortality than patients with TTR‐CMP.

In AL‐CMP patients BNP level was a significant predictor of mortality. Importantly, once female patients are treated with high‐dose melphalan and autologous stem‐cell transplantation, female sex predicts a good outcome in patients who respond to therapy.^36^ Although not statistically significant, TIMP‐1 level showed a tendency to be a predictor in cardiac amyloidosis. In community‐based populations with nonamyloid HF, plasma TIMP‐1 levels were directly associated with LV mass and inversely associated with LV systolic function.^37^ Others have also shown that TIMP‐1 level correlated with echocardiographic parameters of LV dysfunction and remodeling after acute myocardial infarction and identified patients at risk of developing subsequent adverse LV remodeling and of having a poor prognosis.^10^ Interestingly, in our study, circulating TIMP‐1 levels were higher in AL‐CMP patients despite less LV hypertrophy and relatively preserved LVEF compared with TTR‐CMP patients, but because of smaller numbers of patients was not able to predict poor prognosis in AL‐CMP.

### Study Limitations

The present study has several limitations. First, there may be a referral bias because most patients were referred to the Amyloid Treatment and Research Program at Boston Medical Center, a major amyloidosis center. Thus, our findings may be influenced by patients presenting in varying phases of disease. Second, the Cox model should be interpreted with caution because of few events. Third, although this is one of the largest series comparing biomarkers of cardiac amyloidosis in AL‐CMP versus TTR‐CMP, the number of subjects is still relatively small. However, this is a unique data set, and systemic amyloidosis with cardiac involvement is a rare disease. Fourth, although differences in medications may affect these markers, the majority of patients with cardiac amyloidosis arriving for their initial evaluation at our center were only on diuretics. There were also no differences between the groups in this study in the distribution of types of medications. Further insights might be gained by therapeutic intervention studies and comparing pre‐ and postintervention. Fifth*,* as with any blood biomarker, MMP‐2, MMP‐9, TIMP‐1, and TIMP‐2 levels serve only as a “marker” of the localized process that is occurring within tissues and is dependent on release from interstitial compartments into circulation. Some MMP and TIMP interactions are tightly regulated at the site of proteolytic targets and are not released into the blood and may not be reflective of local tissue concentrations. Thus, circulating levels may not be proportional to local interstitial levels or activity. However, our prior work showed that MMP‐9, MMP‐2, and TIMP‐1 were reflective of what is happening at the cardiac level in cardiac amyloidosis.^7^ Finally, these data were correlative and their use for prognosticating has not been tested prospectively.

### Future Directions

In the present era of evidence‐based therapies, for the majority of cardiac amyloid patients, particularly those with restrictive cardiac physiology and autonomic neuropathy, the use of β‐blockers or ACE inhibitors for the treatment of HF is proscribed. In fact, those medications are contraindicated in AL‐CMP patients because they will precipitate clinical deterioration.^38^ Similarly, digoxin induces toxicity by binding to the amyloid fibrils.^39^ Thus, the mainstay of treatment remains diuretics for symptomatic relief. Occasionally spironolactone is used as a K^+^‐sparing diuretic. Importantly, spironolactone is recommended guideline therapy in nonamyloid systolic HF patients and has been shown to improve survival,^40^ cardiac remodeling, and alter the ECM.^41^ Currently, an NIH clinical trial is underway to evaluate spironolactone in nonamyloid diastolic HF (clinicaltrials.gov: NCT00094302). Although high‐dose melphalan and stem‐cell transplant are the therapy to treat amyloidosis in AL‐CMP, persistent and residual LVH increases the propensity toward diastolic dysfunction and heart failure. AL‐CMP patients may present with either diastolic HF (HF with preserved EF) or systolic HF (HF‐reduced EF).

MMP‐9 and TIMP‐1 levels have been associated with LV diastolic dysfunction in a small cohort of AL‐CMP patients.^7^ In vitro studies demonstrate that light chains induce increased ROS in cardiomyocytes.^20–21^ ROS in turn dysregulates MMP activities in cardiomyocytes.^42^ Furthermore, in isolated adult cardiomyocytes, spironolactone modulates ROS and MMP‐2 and MMP‐9 activities.^42^ Given these findings, spironolactone and other novel therapeutic interventions may modulate the ECM and LVH in AL‐CMP patients, and the present biomarker profile could be used as an indication of efficacy. Thus, a clinical trial is sorely needed to allow for future directed therapeutic interventions for AL‐CMP, a rare disease with a dismal prognosis.

In conclusion, our study demonstrates that cardiac amyloidosis patients with immunoglobulin light chain in the heart (AL‐CMP), despite less LVH and relatively preserved LVEF, have higher levels of MMP‐2, TIMP‐1, and MMP‐2/TIMP‐2, as well as increased BNP and TnI compared with patients with transthyretin deposition in the heart (TTR‐CMP). These biomarkers in aggregate (MMP‐2/TIMP‐2 in combination with BNP and TnI) have a potential discriminative ability for distinguishing patients with AL‐CMP from those with TTR‐CMP, allowing for increased diagnostic vigilance with the gold standard—bone marrow biopsy. This may then permit prompt institution of therapy for AL‐CMP. Furthermore, the addition of biomarkers to conventional risk factors for cardiac amyloidosis increases the ability to classify risk, as measured by the c‐statistic, and possibly prognosis.
